# Minimal Clinically Important Difference in Parkinson's Disease as Assessed in Pivotal Trials of Pramipexole Extended Release

**DOI:** 10.1155/2014/467131

**Published:** 2014-04-01

**Authors:** Robert A. Hauser, Mark Forrest Gordon, Yoshikuni Mizuno, Werner Poewe, Paolo Barone, Anthony H. Schapira, Olivier Rascol, Catherine Debieuvre, Mandy Fräßdorf

**Affiliations:** ^1^University of South Florida, Parkinson's Disease and Movement Disorders Center, Byrd Institute, National Parkinson Foundation Center of Excellence, Tampa, FL 33613, USA; ^2^Boehringer Ingelheim Pharmaceuticals, Inc., Ridgefield, CT 06877, USA; ^3^Department of Neurology, Juntendo University School of Medicine, Tokyo 113-8421, Japan; ^4^Department of Neurology, Innsbruck Medical University, Innsbruck, Austria; ^5^Center for Neurodegenerative Diseases, Department of Medicine and Surgery, University of Salerno, Salerno, Italy; ^6^Department of Clinical Neurosciences, University College London Institute of Neurology, London, UK; ^7^Clinical Investigation Center, INSERM CIC-9302 and UMR-825 and Departments of Clinical Pharmacology and Neurosciences, Toulouse University Hospital, Toulouse, France; ^8^Boehringer Ingelheim France S.A.S., Reims, France; ^9^Boehringer Ingelheim Pharmaceuticals, GmbH & Co. KG, Ingelheim, Germany

## Abstract

*Background*. The minimal clinically important difference (MCID) is the smallest change in an outcome measure that is meaningful for patients. *Objectives*. To calculate the MCID for Unified Parkinson's Disease Rating Scale (UPDRS) scores in early Parkinson's disease (EPD) and for UPDRS scores and “OFF” time in advanced Parkinson's disease (APD). *Methods*. We analyzed data from two pivotal, double-blind, parallel-group trials of pramipexole ER that included pramipexole immediate release (IR) as an active comparator. We calculated MCID as the mean change in subjects who received active treatment and rated themselves “a little better” on patient global impression of improvement (PGI-I) minus the mean change in subjects who received placebo and rated themselves unchanged. *Results*. MCIDs in EPD (pramipexole ER, pramipexole IR) for UPDRS II were −1.8 and −2.0, for UPDRS III −6.2 and −6.1, and for UPDRS II + III −8.0 and −8.1. MCIDs in APD for UPDRS II were −1.8 and −2.3, for UPDRS III −5.2 and −6.5, and for UPDRS II + III −7.1 and −8.8. MCID for “OFF” time (pramipexole ER, pramipexole IR) was −1.0 and −1.3 hours. *Conclusions*. A range of MCIDs is emerging in the PD literature that provides the basis for power calculations and interpretation of clinical trials.

## 1. Introduction

Large randomized clinical trials can demonstrate statistically significant differences on outcome measures that may be small and of uncertain relevance as to whether patients actually feel improved [1–3]. The Movement Disorder Society Task Force for Rating Scales for Parkinson's Disease encouraged the identification of a threshold, or the smallest difference on the Unified Parkinson's Disease Rating Scale (UPDRS), that represents the “minimal clinically relevant difference” [4]. The US Food and Drug Administration also expressed the need to determine minimally important differences on measures used to support the labelling claims of medical products [5].

Several investigators have reported assessments of the minimal clinically important difference (MCID) in PD [2, 3, 6, 7]. Although the methodology varied, most studies assessed MCID based on mean change in UPDRS scores in patients defined as minimally improved compared to baseline using the Clinician-rated Global Impression of Improvement (CGI-I) scale. There is a paucity of MCID data based on a patient-rated tool, such as the Patient-rated Global Impression of Improvement (PGI-I). Most of the published MCID data focus on patients with early PD and come from clinical trials of ropinirole or rasagiline [3, 6], but not pramipexole.

Using both PGI-I and CGI-I as anchors, we describe MCID data from two placebo-controlled studies of pramipexole extended release (ER) in patients with early PD (EPD) and advanced PD (APD). Both studies used pramipexole immediate release (IR) as the active comparator. Based on these data, we present several novel findings, not previously explored, including (1) MCID for UPDRS scores in APD, (2) “substantial clinical differences” for UPDRS scores in EPD, and UPDRS scores and “OFF” time for APD, (3) evaluation of the symmetry between calculated minimal important improvement and minimal important worsening, and (4) correlations between PGI-I/CGI-I and changes in UPDRS scores and OFF times.

## 2. Methods

We analyzed data from two pivotal, double-blind, double-dummy, placebo-controlled, parallel-group trials of pramipexole ER. One study (248.524, clinical trial identifier number NCT00479401 at ClinicalTrials.gov) [8, 9] was conducted in subjects with EPD who were Hoehn and Yahr (H-Y) stage 1–3, had disease duration ≤ 5 years, were at least 30 years old at the time of diagnosis, had reached a level of disability requiring initiation of dopaminergic therapy, and were not receiving levodopa or dopamine agonists. The other study (248.525, clinical trial identifier number NCT00466167 at ClinicalTrials.gov) [10] was conducted in subjects with APD who were H-Y stage 2–4 during “on” time, were diagnosed ≥ 2 years before entry, were at least 30 years old, were receiving a stable regimen of levodopa at an optimized dosage, and were experiencing motor fluctuations with at least 2 hours of “OFF” time per day. In both studies, additional antiparkinsonian medications other than dopamine agonists and levodopa were permitted provided that the dosages were stable. In each study, subjects were randomized to treatment with placebo, pramipexole ER, or pramipexole IR. In the EPD study, the randomization ratio was 1 : 2 : 2 and the treatment duration was 33 weeks. In the APD study, the randomization was 1 : 1 : 1 and the treatment duration was 18 weeks. In both studies, the daily dosage of double-blind study medication was up-titrated as tolerated until a response was reached that was judged by the investigator to be at least satisfactory and the subject rated himself at least “a little better,” or until the maximum tolerated or allowed dosage (4.5 mg/day) was reached. Subjects then entered a maintenance phase in which the dosage remained unchanged until the end of trial.

In the EPD study, 539 subjects were randomized. Two primary outcomes were demonstrated: superiority of pramipexole ER over placebo after 18 weeks of treatment and noninferiority between pramipexole ER and IR after 33 weeks of treatment. The primary outcome measure was the change from baseline in UPDRS parts II + III, and secondary outcome measures included responder rates based on PGI-I and CGI-I. Details have been published elsewhere [8, 9].

In the APD trial, 517 subjects were randomized. The primary outcome (change from baseline in UPDRS II + III) demonstrated superiority of pramipexole ER over placebo after 18 weeks of treatment. Secondary outcome measures included change in OFF time based on patient diaries and responder rates based on PGI-I and CGI-I. Details are available elsewhere [10].

For both studies, we analyzed efficacy outcome data from the population of all subjects who received study medication and had a postbaseline efficacy assessment with last observation carried forward for missing data. For the EPD study, mean changes in UPDRS scores were calculated for each PGI-I and CGI-I score at 33 weeks. Changes from baseline were considered separately for UPDRS parts II, III, and II + III. For the APD study, mean changes in OFF time and UPDRS scores were calculated for each PGI-I and CGI-I score at 18 weeks. Changes from baseline were considered separately for UPDRS parts II (mean of UPDRS part II ON and OFF), III, and II + III. In accordance with Hauser and Auinger's previously published methodology [3], we calculated MCID as the mean change in subjects who received active treatment and rated themselves “a little better” minus the mean change in subjects who received placebo and rated themselves unchanged. We also analyzed correlations between CGI-I/PGI-I and change in UPDRS parts II, III, and II + III in the EPD and APD studies and between CGI-I/PGI-I and change in OFF time in the APD study using Spearman correlation coefficients.

The early and advanced trials were conducted between May 2007 and November 2008 at 94 and 76 centers worldwide, respectively [8–10]. Appropriate institutional review boards and ethics committees approved the studies, and all patients provided written informed consent.

## 3. Results

Mean changes in UPDRS II, III, and II + III scores, corresponding to each of the PGI-I and CGI-I ratings for each treatment group, are reported in Table 1 for the EPD study and Table 2 for the APD study. In the EPD study, for subjects rating themselves “a little better,” mean changes in UPDRS II, III, and II + III scores in the pramipexole ER group were −2.4, −7.9, and −10.3 and in the pramipexole IR group −2.6, −7.8, and −10.4. Across a range of PGI ratings, mean changes in UPDRS II + III scores for the pramipexole ER group were −9.0 for “very much better,” −14.4 for “much better,” −10.3 for “a little better,” −8.9 for “no change,” −2.9 for “a little worse,” and 1.3 for “much worse.” In the pramipexole IR group, mean changes in UPDRS II + III scores were −19.0 for “very much better,” −12.6 for “much better,” −10.4 for “a little better,” −6.3 for “no change,” −1.9 for “a little worse,” and 2.5 for “much worse.” Only one subject taking pramipexole ER and no subject taking pramipexole IR self-rated as “very much worse.” Mean changes in UPDRS II, III, and II + III scores for placebo-treated subjects who rated themselves unchanged were −0.6, −1.7, and −2.3.

In the APD study, for subjects rating themselves “a little better,” mean changes in UPDRS II, III, and II + III scores in the pramipexole ER group were −2.8, −8.2, and −11.1 and in the pramipexole IR group were −3.3, −9.5, and −12.8. Across a range of PGI ratings, mean changes in UPDRS II + III scores for the pramipexole ER group were −16.4 for “very much better,” −15.8 for “much better,” −11.1 for “a little better,” −9.3 for “no change,” and −7.8 for “a little worse.” In the pramipexole IR group, changes in UPDRS II + III scores were −23.2 for “very much better,” −16.8 for “much better,” −12.8 for “a little better,” −9.2 for “no change,” −5.2 for “a little worse,” and 1.9 for “much worse.” Only one subject taking pramipexole ER self-rated as “much worse” and one as “very much worse.” Only four subjects taking pramipexole IR self-rated as “much worse” and none as “very much worse.” Mean changes in UPDRS II, III, and II + III scores for placebo-treated subjects who rated themselves unchanged were −1.0, −3.0, and −4.0. Mean change in OFF time for subjects rating themselves “a little better” was −2.0 hours in the pramipexole ER group and −2.3 hours in the pramipexole IR group (Table 3). Mean change in OFF time in placebo-treated subjects who rated themselves unchanged was −1.0 hour.

In the EPD trial, we calculated MCIDs in the pramipexole ER group to be −1.8 for UPDRS II, −6.2 for UPDRS III, and −8.0 for UPDRS II + III and in the pramipexole IR group −2.0 for UPDRS II, −6.1 for UPDRS III, and −8.1 for UPDRS II + III (Table 4). In the APD trial, we calculated MCIDs in the pramipexole ER group to be −1.8 for UPDRS II, −5.2 for UPDRS III, and −7.1 for UPDRS II + III and in the pramipexole IR group −2.3 for UPDRS II, −6.5 for UPDRS III, and −8.8 for UPDRS II + III. In the APD trial, we calculated the MCID for OFF time to be −1.0 hours in the pramipexole ER group and −1.3 hours in the pramipexole IR group.

Spearman correlation coefficients between PGI-I/CGI-I and changes in UPDRS scores and OFF time are presented in Table 5.

## 4. Discussion

Most studies to date have assessed MCID based on change in mean UPDRS scores in subjects rated minimally improved compared to baseline using CGI-I data. We believe that data derived using patient-rated self-impression of change (PGI-I) are more relevant than data derived using clinician-rated impression of change (CGI-I) because we are interested in whether subjects themselves actually feel improved. In our study, using PGI-I or CGI-I scores as the anchor yielded mostly similar results, but there were a few notable exceptions. For example, in the EPD study in the pramipexole ER group, the mean change in UPDRS II + III scores in subjects who rated themselves unchanged was −8.9 whereas the change was −3.3 in subjects who were rated unchanged by clinicians. It is possible that some of these differences could be due to differences in the wording used in the PGI-I compared to the CGI-I (“a little better” versus “minimally improved”); however, it is also possible that a larger UPDRS improvement is required for patients to feel better than for clinicians to observe improvement. Alternatively, and probably more likely, these differences might reflect rater bias in performing UPDRS scoring, with apparent improvement in UPDRS scores being recorded in subjects who are actually little changed.

In general, our data reflect greater improvement in UPDRS scores with better PGI-I and CGI-I ratings. One exception occurs in the EPD study for subjects assigned to pramipexole ER who rated themselves very much improved. We note that this category included a relatively small proportion of subjects (*n* = 10, 5%). Nonetheless, one might hypothesize that these subjects experienced a strong placebo effect (for PGI-I) on top of their “actual” response or that they experienced substantial benefit in areas not adequately captured by the UPDRS. The same effect is seen with CGI-I, but the amplitude is not as great as with PGI-I, which suggests that the PGI-I may have influenced the CGI-I as these are often highly correlated. However, smaller UPDRS improvements were not observed in the patients assigned to pramipexole IR in the EPD study or in either pramipexole group in the APD study. This suggests that this finding may just be spurious or possibly related to an inexact distinction between the two highest PGI-I/CGI-I categories.

Hauser and Auinger [3] previously suggested that if the mean change in the efficacy outcome measure being examined is sufficiently far from zero in placebo-treated subjects who rated themselves as unchanged, one should subtract this “placebo effect” from observed changes in active treatment groups when calculating clinically important differences. Therefore, one can define the MCID as the mean change in the outcome measure in active treatment group subjects who rate themselves “a little better” minus the mean change in placebo-treated subjects who rate themselves as unchanged. For example, in a rasagiline APD trial [3], rasagiline-treated subjects who rated themselves minimally improved recorded a reduction in “OFF” time of 1.9 hours on diaries, whereas placebo-treated subjects who rated themselves as unchanged recorded a reduction in “OFF” time of 0.9 hours, thus yielding a MCID of 1.0 hour (1.9–0.9 hours).

Using this methodology, the MCIDs observed in the EPD trial (pramipexole ER, pramipexole IR) for UPDRS II were −1.8 and −2.0, for UPDRS III −6.2 and −6.1, and for UPDRS II + III −8.0 and −8.1. In a similar analysis of data from a double-blind, prospective study of rasagiline in EPD (week 14), the MCID for UPDRS I + II + III was −3.8 [3]. This is a smaller improvement than our result (for UPDRS II + III) of approximately −8.0. This “discrepancy” is consistent with the notion that the more efficacious the treatment under study, the greater the calculated MCID, likely due to differences in the distribution of outcomes. Schrag et al. [6] evaluated data from two clinical trials of ropinirole IR in EPD based on the criterion of “minimally improved” versus baseline on the CGI-I and reported minimal clinically important change (MCIC) figures very similar to ours (UPDRS II  - 2-3 points, UPDRS III  - 5 points, and total UPDRS  - 8 points) despite the fact that these figures were derived from 6-month studies comparing ropinirole to levodopa or bromocriptine in which there were no placebo control groups and medication dosages were being escalated over time.

We provide calculated “substantial clinical differences (SCDs),” defined as the mean change in the outcome measure in active treatment group subjects who rated themselves “much better” minus the mean change in placebo-treated subjects who rated themselves as unchanged in Table 4. For early PD, our SCDs are in general agreement with those of Shulman et al. [2] for moderate and large differences as derived in a cross-sectional observational analysis of patients with all stages of PD using a variety of clinician- and patient-reported external standards. Table 4 also displays MCIDs and SCDs for advanced PD. We are not aware of other published reports of MCIDs and SCDs in advanced PD but note that in the pramipexole APD study the primary outcome variable was the change in UPDRS II + III, making MCID and SCD for UPDRS scores in APD of interest.

In APD, we found MCIDs for OFF time (pramipexole ER, pramipexole IR) of −1.0 and −1.3 hours. These are similar to the MCID figure of −1.0 hour that was derived using data from a randomized, controlled trial of rasagiline in APD [3]. In the pramipexole APD study, we found SCD for OFF time (pramipexole ER, pramipexole IR) to be −1.5 and −2.2 hours. In this case, the figures derived from the pramipexole ER and pramipexole IR groups are somewhat different, but still in the same general range.

Our data indicate that there is asymmetry regarding clinically important change for improvement versus worsening. For example, in the EPD trial, for subjects assigned to pramipexole ER who rated themselves “a little better,” UPDRS III scores improved by 7.9 points. Subjects who rated themselves “a little worse” did not experience a UPDRS worsening of 7.9 points but instead were rated improved by 3.0 points. If the change in the group of placebo subjects who rated themselves unchanged is subtracted from these figures, one finds that while the MCID for improvement (for UPDRS III in the pramipexole ER group) is an improvement of 6.2 points, the MCID for worsening is an improvement of 1.3 points. This asymmetry probably reflects the distribution of change in UPDRS scores in the treated population based on both placebo and treatment effects. The direction and degree of asymmetry probably depend on both expectation (placebo effect) and the actual efficacy of the intervention. Therefore, clinical trials of effective medications cannot be used to determine how much deterioration patients in clinical practice would need to experience over time to consider themselves meaningfully worse.

As anticipated, a range of values for MCID for improvement in EPD based on UPDRS scores is emerging. Differences in outcome appear to depend on many factors including the original study design, efficacy of the intervention, and the anchor selected. We believe the most relevant results come from trials that include a placebo control group, test interventions of mild efficacy, and depend on patient-reported outcomes such as the PGI-I. Interestingly, we found that the MCID for improvement in OFF time in the APD trial was a reduction of 1.0–1.3 hours, similar to what was observed using data from a rasagiline APD trial. Whether all studies in APD will yield similar values or whether a range will emerge based on the efficacy of the intervention remains to be seen. Additional analyses are needed from trials of highly efficacious interventions such as deep brain stimulation (DBS).

Correlations between CGI-I/PGI-I and UPDRS scores and OFF time were mostly in the moderate range. Interestingly, for both EPD and APD, the strongest correlations were between the CGI-I and UPDRS III and UPDRS II + III. This may reflect the fact that the CGI-I and UPDRS III scores are both clinician-rated. These ratings may reflect the physical appearance of the patient but may fail to account for other factors important to patients, such as nonmotor features and adverse events. Rater bias may also contribute to this effect and potentially explain why in some cases UPDRS scores suggest improvement while PGI scores indicate worsening.

The methodology used to determine MCID is imperfect and studies have provided a range of values rather than a single value. Nonetheless, MCID is useful to perform power calculations and to understand the meaning of the magnitude of change one observes in a clinical trial. In the pramipexole ER pivotal trials, MCIDs in subjects with EPD (pramipexole ER, pramipexole IR) for UPDRS II were −1.8 and −2.0, for UPDRS III −6.2 and −6.1, and for UPDRS II + III −8.0 and −8.1. MCIDs in subjects with APD for UPDRS II were −1.8 and −2.3, for UPDRS III −5.2 and −6.5, and for UPDRS II + III −7.1 and −8.8. In the APD study, we found that the MCID for OFF time (pramipexole ER, pramipexole IR) was −1.0 and −1.3 hours. The current study provides support for the hypothesis [3] that more efficacious interventions yield larger calculated MCIDs. The difference in mean change in UPDRS II + III scores for pramipexole ER compared to placebo in EPD was −7.0 [9] and the calculated MCID was −8.0 whereas the adjusted effect size for change in UPDRS I + II + III for rasagiline compared to placebo was −4.2 and the calculated MCID was −3.8 [3, 11]. The lower end of the range of MCIDs emerging from clinical trials may represent a threshold effect size that should be met to conclude that an intervention has a meaningful clinical impact. In surveying the literature to date, studies suggest that the lower limit of MCID for total UPDRS (I + II + III) is −3.8 and for change on OFF time is −1.0 hour [3].

## Figures and Tables

**Table 1:** Mean changes in UPDRS II, III, and II+III scores according to PGI-I and CGI-I ratings by treatment group in the early PD study.

PGI-I	*n* (%)	UPDRS II	UPDRS III	UPDRS II+III	CGI-I	*n* (%)	UPDRS II	UPDRS III	UPDRS II+III
Pramipexole ER									
Very much better	10 (5%)	−3.5 (3.3)	−5.5 (4.9)	−9.0 (6.4)	Very much improved	15 (7%)	−2.7 (3.8)	−8.1 (7.6)	−10.9 (10.2)
Much better	63 (30%)	−3.6 (3.0)	−10.7 (8.4)	−14.4 (10.0)	Much improved	76 (38%)	−3.8 (3.1)	−11.3 (8.5)	−15.1 (10.2)
A little better	59 (28%)	−2.4 (3.3)	−7.9 (8.4)	−10.3 (10.6)	Minimally improved	71 (34%)	−2.5 (2.7)	−7.0 (5.7)	−9.4 (7.1)
No change	45 (21%)	−2.6 (2.8)	−6.3 (5.7)	−8.9 (7.4)	No change	33 (16%)	−0.9 (2.4)	−2.3 (5.4)	−3.3 (6.8)
A little worse	22 (10%)	0.1 (3.0)	−3.0 (4.9)	−2.9 (6.9)	Minimally worse	12 (6%)	1.9 (3.3)	1.5 (6.8)	3.4 (8.8)
Much worse	12 (6%)	0.8 (3.7)	0.6 (6.7)	1.3 (9.4)	Much worse	3 (1%)	2.7 (5.5)	2.3 (5.7)	5.0 (10.5)
Very much worse	1 (0.5%)	0	7	7	Very much worse	0			

Pramipexole IR									
Very much better	8 (4%)	−5.4 (2.6)	−13.6 (7.5)	−19.0 (9.6)	Very much improved	14 (7%)	−5.5 (2.5)	−14.1 (8.1)	−19.6 (8.9)
Much better	61 (29%)	−3.8 (3.7)	−8.8 (8.3)	−12.6 (10.7)	Much improved	81 (39%)	−3.9 (3.6)	−10.3 (8.5)	−14.1 (10.6)
A little better	73 (35%)	−2.6 (3.3)	−7.8 (8.5)	−10.4 (10.1)	Minimally improved	66 (32%)	−2.1 (3.1)	−5.6 (6.7)	−7.7 (8.2)
No change	44 (21%)	−1.6 (2.9)	−4.8 (8.3)	−6.3 (9.9)	No change	34 (17%)	−0.5 (2.3)	−1.9 (6.8)	−2.4 (7.9)
A little worse	17 (8%)	0.0 (3.0)	−1.9 (5.5)	−1.9 (7.8)	Minimally worse	9 (4%)	0.0 (2.8)	1.6 (5.6)	1.6 (7.2)
Much worse	4 (2%)	0.3 (3.3)	2.3 (4.8)	2.5 (7.0)	Much worse	2 (1%)	3.0 (1.4)	3.5 (2.1)	6.5 (0.7)
Very much worse	0				Very much worse	0			

Placebo									
Very much better	4 (4%)	−1.8 (3.0)	−7.0 (6.7)	−8.8 (6.4)	Very much improved	2 (2%)	−2.5 (3.5)	−6.0 (4.2)	−8.5 (7.8)
Much better	18 (18%)	−3.4 (2.8)	−6.7 (5.9)	−10.1 (6.9)	Much improved	28 (28%)	−2.3 (2.8)	−6.8 (4.4)	−9.1 (5.1)
A little better	34 (33%)	−1.0 (3.5)	−5.2 (8.4)	−6.2 (10.2)	Minimally improved	31 (30%)	−1.3 (3.4)	−7.3 (7.6)	−8.5 (9.0)
No change	34 (33%)	−0.6 (3.2)	−1.7 (7.1)	−2.3 (8.8)	No change	29 (28%)	−0.5 (3.5)	0.7 (5.7)	0.2 (7.9)
A little worse	10 (10%)	1.7 (3.7)	1.8 (3.3)	3.5 (6.3)	Minimally worse	11 (11%)	1.8 (4.1)	4.1 (6.1)	5.9 (9.5)
Much worse	3 (3%)	0.3 (2.3)	5.3 (2.5)	5.7 (4.7)	Much worse	1 (1%)	2	13	15
Very much worse	0				Very much worse	0			

Presented as the mean change in the UPDRS scores (standard deviations).

PGI-I: Patient-rated Global Impression of Improvement; CGI-I: Clinician-rated Global Impression of Improvement.

**Table 2:** Mean changes in UPDRS II, III, and II+III scores according to PGI-I and CGI-I ratings by treatment group in the advanced PD study.

PGI-I	*n* (%)	UPDR II	UPDRS III	UPDRS II+III	CGI-I	*n* (%)	UPDRS II	UPDRS III	UPDRS II+III
Pramipexole ER									
Very much better	9 (6%)	−3.7 (2.5)	−12.7 (5.5)	−16.4 (7.2)	Very much improved	9 (6%)	−4.3 (2.5)	−13.3 (6.6)	−17.7 (8.8)
Much better	51 (32%)	−4.6 (4.3)	−11.2 (12.7)	−15.8 (15.9)	Much improved	69 (43%)	−4.3 (4.4)	−11.2 (11.3)	−15.5 (14.4)
A little better	66 (41%)	−2.8 (3.8)	−8.2 (8.8)	−11.1 (11.2)	Minimally improved	51 (32%)	−2.0 (3.9)	−7.7 (9.3)	−9.7 (11.9)
No change	20 (12%)	−2.5 (4.0)	−6.8 (9.8)	−9.3 (12.5)	No change	22 (14%)	−3.3 (3.6)	−7.8 (6.6)	−11.1 (7.9)
A little worse	13 (8%)	−0.9 (4.4)	−6.9 (6.4)	−7.8 (8.2)	Minimally worse	7 (4%)	−0.5 (2.7)	1.6 (9.7)	1.1 (10.2)
Much worse	1 (0.6%)	−1.0	6.0	5.0	Much worse	2 (1%)	0.3 (6.7)	−1.0 (0.0)	−0.8 (6.7)
Very much worse	1 (0.6%)	5.0	−1.0	4.0	Very much worse	0			

Pramipexole IR									
Very much better	10 (6%)	−6.3 (3.5)	−16.9 (12.9)	−23.2 (13.9)	Very much improved	11 (7%)	−5.4 (3.4)	−14.5 (7.1)	−20.0 (6.8)
Much better	66 (38%)	−4.9 (4.2)	−11.9 (9.9)	−16.8 (12.0)	Much improved	77 (46%)	−5.2 (4.3)	−12.9 (10.4)	−18.0 (12.6)
A little better	55 (32%)	−3.3 (4.1)	−9.5 (9.8)	−12.8 (12.9)	Minimally improved	55 (33%)	−3.0 (3.5)	−8.4 (9.0)	−11.3 (11.4)
No change	27 (16%)	−34.1 (4.2)	−6.0 (6.4)	−9.2 (8.6)	No change	19 (11%)	−2.5 (4.0)	−2.8 (7.4)	−5.3 (9.7)
A little worse	10 (6%)	−2.0 (2.4)	−3.2 (10.4)	−5.2 (10.9)	Minimally worse	4 (2%)	−0.6 (5.0)	−6.0 (3.6)	−6.6 (8.0)
Much worse	4 (2%)	0.1 (6.0)	1.8 (10.1)	1.9 (15.8)	Much worse	3 (2%)	−0.5 (7.1)	2.3 (9.3)	1.8 (16.1)
Very much worse	0				Very much worse	0			

Placebo									
Very much better	3 (2%)	−8.3 (8.8)	−11.0 (9.5)	−19.3 (18.3)	Very much improved	6 (4%)	−6.4 (6.3)	−9.5 (6.8)	−15.9 (13.1)
Much better	44 (26%)	−4.0 (5.8)	−8.9 (13.8)	−12.9 (18.6)	Much improved	50 (29%)	−3.8 (5.4)	−9.8 (12.1)	−13.6 (16.3)
A little better	65 (37%)	−1.9 (3.6)	−3.8 (7.2)	−5.6 (8.6)	Minimally improved	66 (39%)	−1.8 (3.7)	−3.8 (9.0)	−5.7 (10.8)
No change	43 (25%)	−1.0 (3.9)	−3.0 (6.7)	−4.0 (9.0)	No change	31 (18%)	−1.1 (4.7)	−1.0 (6.0)	−2.1 (7.9)
A little worse	12 (7%)	0.1 (4.5)	−1.3 (13.5)	−1.1 (15.6)	Minimally worse	15 (9%)	0.0 (3.3)	0.9 (6.2)	1.0 (7.9)
Much worse	7 (4%)	0.1 (5.6)	−1.1 (3.9)	−1.1 (8.5)	Much worse	3 (2%)	5.3 (4.0)	3.0 (4.4)	8.3 (8.3)
Very much worse	0				Very much worse	0			

Presented as the mean change in the UPDRS scores (standard deviations).

PGI-I: Patient-rated Global Impression of Improvement; CGI-I: Clinician-rated Global Impression of Improvement.

**Table 3:** Mean changes in OFF time according to PGI-I and CGI-I ratings by treatment group in the advanced PD study.

PGI-I	*n* (%)	OFF time hrs (S.D.)	CGI-I	*n* (%)	OFF time hrs (S.D.)
Pramipexole ER					
Very much better	9 (6%)	−2.9 (2.5)	Very much improved	9 (6%)	−2.8 (2.2)
Much better	51 (33%)	−2.5 (3.2)	Much improved	69 (41%)	−2.7 (3.2)
A little better	66 (41%)	−2.0 (2.8)	Minimally improved	51 (32%)	−1.7 (2.8)
No change	20 (13%)	−1.3 (2.9)	No change	22 (14%)	−1.0 (3.0)
A little worse	13 (8%)	−0.6 (3.9)	Minimally worse	7 (4%)	1.3 (3.6)
Much worse	1 (0.6%)	5.3	Much worse	1 (0.6%)	0.3
Very much worse	0		Very much worse	0	

Pramipexole IR					
Very much better	10 (6%)	−4.4 (1.6)	Very much improved	11 (7%)	−3.6 (2.7)
Much better	66 (39%)	−3.2 (2.6)	Much improved	77 (46%)	−3.3 (2.5)
A little better	55 (32%)	−2.3 (2.2)	Minimally improved	55 (33%)	−2.1 (2.2)
No change	27 (16%)	−1.2 (2.7)	No change	19 (11%)	−0.9 (2.8)
A little worse	10 (6%)	−1.4 (3.8)	Minimally worse	4 (2%)	0.5 (4.2)
Much worse	3 (2%)	2.3 (5.6)	Much worse	3 (2%)	2.7 (5.3)
Very much worse	0		Very much worse	0	

Placebo					
Very much better	3 (2%)	−5.1 (3.6)	Very much improved	6 (4%)	−4.4 (3.2)
Much better	44 (25%)	−1.9 (3.1)	Much improved	50 (29%)	−2.3 (2.2)
A little better	65 (37%)	−1.5 (3.4)	Minimally improved	66 (39%)	−0.9 (2.9)
No change	43 (25%)	−1.0 (2.2)	No change	31 (18%)	−1.0 (2.3)
A little worse	12 (7%)	0.3 (3.6)	Minimally worse	15 (9%)	0.0 (3.6)
Much worse	7 (4%)	−0.5 (2.1)	Much worse	3 (2%)	−1.1 (2.9)
Very much worse	0		Very much worse	0	

Presented as the mean change in OFF time (standard deviations).

PGI-I: Patient-rated Global Impression of Improvement; CGI-I: Clinician-rated Global Impression of Improvement.

**Table 4:** The minimal clinically important difference and the substantial clinical difference in UPDRS scores in early PD and advanced PD.

Change	Pramipexole	Early PD (PGI-I data)			Advanced PD (PGI-I data)			
		UPDRS II	UPDRS III	UPDRS II+III	UPDRS II	UPDRS III	UPDRS II+III	OFF time (hrs)
Minimal clinically important difference	Extended release	−1.8	−6.2	−8.0	−1.8	−5.2	−7.1	−1.0
	Immediate release	−2.0	−6.1	−8.1	−2.3	−6.5	−8.8	−1.3

Substantial clinical difference	Extended release	−3.0	−9.0	−12.1	−3.6	−8.2	−11.8	−1.5
	Immediate release	−3.2	−7.1	−10.3	−3.9	−8.9	−12.8	−2.2

PGI-I: Patient-rated Global Impression of Improvement.

(i) Minimal clinically important difference is defined as the mean change in the outcome measure in active treatment group subjects who rated themselves “a little better” on PGI-I minus the mean change in placebo-treated subjects who rated themselves as unchanged.

(ii) Substantial clinical difference is defined as the mean change in the outcome measure in active treatment group subjects who rated themselves “much better” on PGI-I minus the mean change in placebo-treated subjects who rated themselves as unchanged.

**Table 5:** Spearman correlation coefficients between PGI-I/CGI-I and changes in UPDRS scores and OFF time in patients with early PD and advanced PD.

Global improvement	Early PD			Advanced PD					
	UPDRS II	UPDRS III	UPDRS II+III	UPDRS II	UPDRS III	UPDRS II+III	UPDRS II ON	UPDRS II OFF	OFF time (hrs)
CGI-I	0.42	0.51	0.54	0.33	0.4	0.42	0.19	0.36	0.31
PGI-I	0.35	0.34	0.39	0.31	0.3	0.34	0.21	0.32	0.27

PGI-I: Patient-rated Global Impression of Improvement; CGI-I: Clinician-rated Global Impression of Improvement.
